# Factors associated with mental health outcomes among medical residents exposed to COVID-19

**DOI:** 10.1192/bjo.2021.12

**Published:** 2021-02-15

**Authors:** Mohamed Adil Shah Khoodoruth, Saleem Khaldoon Al-Nuaimi, Zerak Al-Salihy, Adeel Ghaffar, Widaad Nuzhah Chut-kai Khoodoruth, Sami Ouanes

**Affiliations:** Psychiatry Residency Program, Hamad Medical Corporation, Qatar; Department of Psychiatry, Hamad Medical Corporation, Qatar; Department of Psychiatry, Hamad Medical Corporation, Qatar; Medical Education Department, Hamad Medical Corporation, Qatar; Centre of Disease Control and Prevention Department, Hamad Medical Corporation, Qatar; Department of Psychiatry, Hamad Medical Corporation, Qatar

**Keywords:** Mental health, healthcare workers, medical residents, COVID-19, pandemic

## Abstract

**Background:**

The COVID-19 outbreak has caused challenges for healthcare systems worldwide. Recent data indicates that the psychological impact has differed with respect to occupation. In many countries, medical residents have been on the front line of this pandemic. However, data on the psychological impact of infectious disease outbreaks, and COVID-19 in particular, on medical residents are relatively lacking.

**Aims:**

The aim of our study was to assess the psychological impact of the COVID-19 pandemic on medical residents working on the front and second line.

**Method:**

An electronic survey was sent to all medical residents in Qatar. Depression, anxiety and stress were assessed by the Depression, Anxiety and Stress Scale – 21 Items. Professional quality of life was measured by the Professional Quality of Life measure.

**Results:**

Of the 640 medical residents contacted, 127 (20%) responded. A considerable proportion of residents reported symptoms of depression (42.5%), anxiety (41.7%) and stress (30.7%). Multivariate analysis of variance showed significant effects of seniority in residency, with junior residents having poorer outcomes. In addition, there was a statistically significant interaction effect with moderate effect sizes between gender and working on the front line, as well as gender, working on the front line and seniority, on mental health outcomes.

**Conclusions:**

The COVID-19 pandemic may have a negative impact on junior residents’ mental health. Preventive measures to reduce stress levels and easy access to professional mental health services are crucial.

Medical residents are doctors in training and account for the majority of patient contacts with doctors within teaching hospitals, and they are, consequently, at risk of exposure to communicable disease.^1^ Previous studies have showed a strikingly high rate of burnout and depression among medical residents: 51.5% of 16 192 internal medicine residents in the largest multicentre study on medical resident burnout, and 28.8% of 17 560 resident physicians reported symptoms of depression in a meta-analysis.^2,3^ Medical residents form a substantial segment of the healthcare staff responding to the COVID-19 pandemic internationally. This pandemic has strikingly changed the lives and outlook of residents in a very short time. For instance, the ‘normal’ daily, weekly, monthly and annual schedules planned by the residency programmes, which were to be in effect at this time, have been heavily affected; for example, deployment of medical residents as primary care doctors in screening facilities; shifting supervision, didactics and educational conferences online; and lack of clinical exposure because of curtailing office visits.^4^ However, data on the psychological effects of infectious disease outbreaks, and COVID-19 in particular, on medical residents are relatively lacking even though the psychological aftermath of the 2003 SARS and COVID-19 outbreaks seem to differ with respect to occupation.^5–7^ A PubMed search on 31 July 2020 for ‘(covid-19 OR coronavirus) AND (medical residents OR trainees)’ identified 194 results. Out of 50 articles that specifically targeted medical residents or residency programmes, nine cross-sectional studies earmarked the mental health outcomes of the COVID-19 pandemic on medical residents.^8–16^ Although South Korean orthopaedic residents have reported a decrease in their quality of life during the pandemic, data on the impact of COVID-19 on medical residents’ mental health outcomes remains scarce, according to our literature search.^10^ Although a nationwide study among otolaryngology residents and attending physicians in the USA indicated a higher level of resident burnout, medical residents in China had a lower risk of psychological problems than other physicians and nurses.^12,15^ Working on the front line and directly engaging in the diagnosis, treatment and care of patients with COVID-19 may also be a key factor. In Wuhan, China, the epicentre of the original outbreak, front-line staff were found to have higher psychological burden than second-line staff.^6^ On the other hand, front-line medical residents in Romania had lower levels of burnout than second-line medical residents.^13^ It is also unclear how seniority in residency affects mental health outcomes during the COVID-19 pandemic.^11^ To address this gap, we aimed to assess levels of symptoms of depression, anxiety and stress, and professional quality of life, among medical residents with the Hamad Medical Corporation (HMC) in Qatar.

## Method

### Setting and participants

We conducted a cross-sectional study among medical residents from all specialties in Qatar. All residency programmes in the country are with HMC, an institution accredited by the Accreditation Council for Graduate Medical Education-International (ACGME-I). A resident is a physician enrolled in an ACGME-accredited residency programme, usually after graduation from the medical school. The residency programme is designed to prepare resident physicians to practice independently in a primary specialty.^17^

The survey took place from 17 May to 16 June 2020. During this period, the total confirmed cases of COVID-19 in Qatar exceeded 80 000, with 80 deaths. HMC, the main public healthcare system in Qatar, is the largest provider of tertiary health services in the country, accounting for more than 90% of the care delivered, with only few and relatively small private healthcare systems.

In our questionnaire we did not include information about how to seek help. However, HMC residency programmes have mentorship programmes and access to counselling through a variety of channels; for example, graduate medical education, trainee council, trainee psychology services and routine mental health services. Even before the pandemic started, and more so after it occurred, the residents have been especially encouraged to get support.

The study was approved by the Hamad Medical Corporation Institutional Review Board (approval number MRC-05-049), and all study participants provided electronic consent.

### Data collection and instruments

The survey was built with Qualtrics software (Qualtrics, Utah, USA). An email was sent to the professional email addresses of all medical residents in the country. To improve the participation rates, reminder emails were sent at 1, 2 and 3 weeks after the initial email.

To measure the mental health outcomes, we used two scales. The Depression, Anxiety and Stress Scale – 21-Items (DASS-21) quantitatively measures distress along the three axes of symptoms of depression, anxiety and stress. Higher scores in any of the axes indicate higher levels of distress/symptoms. The suggested cut-off scores for detecting symptoms of major depression, anxiety and stress are 4, 3 and 7, respectively.^18^

The Professional Quality of Life measure (ProQOL) is the most commonly used measure of the positive and negative effects of working with people who have experienced extremely stressful events.^19^ It measures compassion satisfaction (a low score (<23) signifies a poor level of professional satisfaction) and compassion fatigue. The latter is broken down into two parts: burnout (higher score (>41) indicating higher risk for burnout) and secondary traumatic stress (a score >43 indicating a frightening experience at work).^19^

### Statistical analyses

All statistical analyses were done with IBM SPSS software version 25 for Windows. For descriptive statistics, we calculated absolute and relative frequencies for categorical variables, and means and s.d. for continuous variables. We used t-test for independent samples to compare the different DASS-21 and ProQOL scores between groups.

To assess the differences in mental health outcomes between groups (senior versus junior residents, and front-line versus second-line staff), we constructed a three-way multivariate analyses of variance (MANOVA), using DASS-21 depression, anxiety and stress scores as dependent variables, with ‘residency seniority’ (junior residents being in their first or second year of training, with senior residents being in their third year or onward), working on the front or second line, and gender as independent variables. A medical resident working on the front line is one who is directly engaged in the diagnosis, treatment and care of patients with COVID-19. Second-line medical residents do not face and address patients with COVID-19. Preliminary assumptions for MANOVA (including normality, linearity, univariate and multivariate outliers, homogeneity of variance, covariance matrices and multicollinearity) were tested. Pillai's trace test was used because the DASS-21 scores violated the normality assumption. The effect size was assessed with partial η^2^. The defined significance level was α = 0.05.

## Results

### Response rates

Out of 640 medical residents contacted, 127 (20%) responded to the survey. The participants could not skip individual items, therefore there were no item-level missing data.

### Sociodemographic characteristics

Table 1 displays the medical residents’ characteristics and mental health outcomes severity categories in the total sample. Six (4.7%) respondents reported having been diagnosed with a psychiatric disorder: four (3.1%) with depression and two (1.6%) with anxiety. Fourteen (11.0%) residents were on chronic medications: four (3.1%) on thyroid replacement therapy, four (3.1%) on antihypertensives and one (0.8%) on fluoxetine.

**Table 1:** Characteristics of residents and severity categories in the total cohort

Characteristic	*n* (%)
Gender, male	79 (63)
Age, years	
25–30	94 (74)
30–35	31 (24)
>35	2 (2)
Marital status	
Single	61 (48)
Married	62 (49)
Others	4 (3)
Parental status	
No children	96 (76)
Have children	31 (24)
Year of residency^a^	
Junior residents	71 (56)
Senior residents	57 (44)
COVID-19 front line, yes	80 (63)
DASS-21	
Depression score	
Normal	73 (57.5)
Mild	16 (12.6)
Moderate	23 (18.1)
Severe	9 (7.1)
Extremely severe	6 (4.7)
Anxiety score	
Normal	74 (58.3)
Mild	21 (16.5)
Moderate	16 (12.6)
Severe	10 (7.9)
Extremely severe	6 (4.7)
Stress score	
Normal	88 (69.3)
Mild	13 (10.2)
Moderate	11 (8.7)
Severe	14 (11.0)
Extremely severe	1 (0.8)
ProQOL	
Compassion satisfaction	
High	119 (93.7)
Low or moderate	8 (6.3)
Burnout	
High	0 (0)
Low or moderate	127 (100)
Secondary traumatic stress	
High	1 (0.8)
Low or moderate	126 (99.2)

DASS-21, Depression, Anxiety and Stress Scale – 21-Items; ProQOL, Professional Quality of Life measure.

a. Junior residents (first 2 years of training) and senior residents (third year onward).

A considerable proportion of residents had symptoms of depression (42.5%), anxiety (41.7%) and stress (30.7%). Overall, most (93.7%) participants had high levels of compassion satisfaction, all had low-to-moderate levels of burnout and virtually all (99.2%) had secondary traumatic stress.

### Severity of measurements and associated factors

Table 2 shows the scores for symptoms of depression, anxiety, stress, compassion satisfaction, burnout and secondary traumatic stress in the total sample and subgroups. The mean scores on the DASS-21 for depression, anxiety and stress for all respondents were 4.8 ± 4.5, 3.6 ± 3.3 and 6.1 ± 4.2, respectively. In the sample, independent t-test comparisons did not show any statistical differences between gender, junior versus senior residents or front-line versus second-line residents. Among women, the junior residents exhibited higher levels of stress (8.1 *v.* 5.5, *P* = 0.046), but depression and anxiety scores were not significantly different. No difference in mental health outcomes was noted between junior and senior male residents.

**Table 2:** Scores of depression, anxiety, stress, compassionate satisfaction and compassionate fatigue measurement in total cohort and subgroups

	Gender			COVID-19 front line			Year of residency^a^		
Scale	Total score, mean (s.d.)	Male	Female	*P*-value	Yes	No	*P*-value	Junior residents	Senior residents	*P*-value
DASS-21										
Depression	4.8 (4.5)	4.8 (4.5)	5.0 (4.6)	0.793	4.7 (4.6)	5.3 (4.5)	0.434	5.5 (5.2)	4.1 (3.5)	0.066
Anxiety	3.6 (3.3)	3.5 (3.2)	3.8 (3.4)	0.591	3.4 (3.2)	3.9 (3.0)	0.494	3.9 (3.5)	3.2 (3.0)	0.209
Stress	6.1 (4.2)	5.9 (4.3)	6.8 (4.2)	0.296	5.9 (4.2)	6.8 (4.3)	0.251	6.9 (4.6)	5.4 (3.7)	0.053
ProQOL										
Compassion satisfaction	36.4 (8.3)	35.4 (8.3)	38.0 (8.1)	0.106	36.7 (8.4)	36.0 (8.2)	0.662	35.8 (8.9)	37.2 (7.5)	0.399
Burnout	25.3 (6.5)	25.5 (6.6)	24.6 (5.9)	0.423	25.0 (6.5)	25.4 (6.3)	0.728	25.8 (6.8)	24.4 (5.8)	0.234
Secondary traumatic stress	22.6 (7.0)	21.7 (6.7)	24.0 (7.1)	0.077	22.3 (7.0)	23.0 (6.9)	0.627	23.3 (7.5)	21.6 (6.0)	0.170

All scores are expressed as mean (s.d.). DASS-21, Depression, Anxiety and Stress Scale – 21-Items; ProQOL, Professional Quality of Life measure.

a. Junior residents (first 2 years of training) and senior residents (third year onward).

The mean scores on the ProQOL for compassion satisfaction, burnout and secondary traumatic stress for all respondents were 36.4 ± 8.3, 25.3 ± 6.5 and 22.6 ± 7.0, respectively. Independent t-test comparisons did not show any statistical differences between gender, junior versus senior residents or front-line versus second-line residents.

The three-way MANOVA analysis examining mental health outcomes (DASS-21 depression, anxiety and stress scores) as dependent variables, with seniority in residency, working on the front line and gender as independent variables, showed that the mental health outcomes were significantly affected by seniority in residency, with junior residents having poorer outcomes. In addition, there was a statistically significant interaction effect with moderate effect sizes between gender and working on the front line, as well as gender, working on the front line and seniority, on the mental health outcomes (Table 3).

**Table 3:** Significance levels of the three-way multiple variance analysis test assessing the associations between mental health outcomes (DASS-21) in medical residents, and seniority, working on the front line and gender

Effect	Pillai's trace	F-value	α	Partial η^2^
Gender	0.019	0.747	0.526	0.019
Front-line worker	0.010	0.391	0.760	0.010
Seniority in residency	0.077	3.246	**0.024**	0.077
Gender×front-line worker	0.097	4.201	**0.007**	0.097
Gender×seniority in residency	0.008	0.297	0.828	0.008
Front-line worker×seniority in residency	0.021	0.857	0.466	0.021
Gender×front-line worker×seniority in residency	0.092	3.952	**0.010**	0.092

DASS-21, Depression, Anxiety and Stress Scale – 21-Items. Values in bold denote statistical significance.

Univariate tests of between-participant effects (Table 4) showed significantly higher DASS-21 depression, anxiety and stress scores among junior residents, as well as significant effects of the gender×front-line worker interaction on DASS-21 depression score (meaning that the effect of gender on the depression score depended on working on the front line versus second line, and vice versa), and significant effects of the gender×front-line worker×seniority interaction on all three scores (with junior female front-line residents, and male junior second-line residents exhibiting the poorest outcomes). *Post-hoc* power analysis, meaning the retrospective power derived from our data-set, showed an observed power of 0.604.

**Table 4:** Univariate tests of between-participant effects, with DASS-21 scores as dependent variables

Source	Dependent variable	Type 3 sum of squares	Mean square	F-value	α	Partial η^2^
Gender	DASS-21 Depression	3.119	3.119	0.169	0.682	0.001
	DASS-21 Anxiety	0.647	0.647	0.063	0.802	0.001
	DASS-21 Stress	7.904	7.904	0.489	0.486	0.004
Front-line worker	DASS-21 Depression	2.883	2.883	0.156	0.694	0.001
	DASS-21 Anxiety	3.130	3.130	0.306	0.581	0.003
	DASS-21 Stress	15.685	15.685	0.971	0.326	0.008
Seniority in residency	DASS-21 Depression	147.735	147.735	7.986	**0.006**	0.063
	DASS-21 Anxiety	51.793	51.793	5.056	**0.026**	0.041
	DASS-21 Stress	144.665	144.665	8.957	**0.003**	0.070
Gender×front-line worker	DASS-21 Depression	109.615	109.615	5.926	**0.016**	0.047
	DASS-21 Anxiety	1.228	1.228	0.120	0.730	0.001
	DASS-21 Stress	0.358	0.358	0.022	0.882	0.000
Gender×seniority in residency	DASS-21 Depression	3.019	3.019	0.163	0.687	0.001
	DASS-21 Anxiety	7.044	7.044	0.688	0.409	0.006
	DASS-21 Stress	0.851	0.851	0.053	0.819	0.000
Front-line worker×seniority in residency	DASS-21 Depression	0.140	0.140	0.008	0.931	0.000
	DASS-21 Anxiety	19.565	19.565	1.910	0.170	0.016
	DASS-21 Stress	4.292	4.292	0.266	0.607	0.002
Gender×front-line worker×seniority in residency	DASS-21 Depression	163.836	163.836	8.857	**0.004**	0.069
	DASS-21 Anxiety	48.217	48.217	4.707	**0.032**	0.038
	DASS-21 Stress	184.846	184.846	11.445	**0.001**	0.088

DASS-21, Depression, Anxiety and Stress Scale – 21-Items. Values in bold denote statistical significance.

Spearman correlations between mental health outcomes (Table 5) showed a very strong positive correlation between stress and anxiety (*ρ* = 0.71, *P* ≤ 0.01). Moreover, there was a very strong negative correlation between burnout and compassion satisfaction (*ρ* = 0.78, *P* ≤ 0.01).

**Table 5:** Spearman correlations between mental health outcomes

		DASS-21 Depression	DASS-21 Anxiety	DASS- 21 Stress	Compassion satisfaction	Secondary traumatic stress
DASS-21 Depression	Correlation coefficient					
	Significance (two-tailed)					
DASS-21 Anxiety	Correlation coefficient	0.592**				
	Significance (two-tailed)	0.000				
DASS-21 Stress	Correlation coefficient	0.693**	0.706**			
	Significance (two-tailed)	0.000	0.000			
Compassion satisfaction	Correlation coefficient	−0.515**	−0.226*	−0.298**		
	Significance (two-tailed)	0.000	0.014	0.001		
Secondary traumatic stress	Correlation coefficient	0.445**	0.539**	0.535**	−0.227*	
	Significance (two-tailed)	0.000	0.000	0.000	0.014	
Burnout	Correlation Coefficient	0.616**	0.359**	0.474**	−0.771**	0.548**
	Sig. (two-\tailed)	0.000	0.000	0.000	0.000	0.000

DASS-21, Depression, Anxiety and Stress Scale – 21-Items.

* *P*<0.05 (two-tailed), ** *P*<0.01 (two-tailed).

**Fig. 1:**
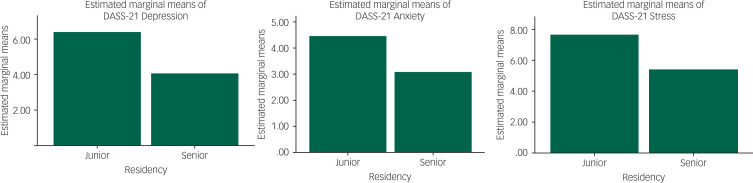
Estimated marginal means of mental health outcomes, using the generalised linear model procedure. DASS-21, Depression, Anxiety and Stress Scale – 21-Items.

Figure 1 shows the estimated marginal means of mental health outcomes, using the univariate generalised linear model feature in SPSS, adjusted for by other variables.

## Discussion

This cross-sectional survey enrolled 127 respondents and revealed a high prevalence of mental health symptoms among the residents during the COVID-19 pandemic in Qatar. Overall, 42.5%, 41.7% and 30.7% all participants reported symptoms of depression, anxiety and stress, respectively. In contrast, the residents demonstrated high compassion satisfaction, moderate-to-low burnout and secondary traumatic stress, all of which translate into high professional quality of life. Multivariate analysis showed significant effects of seniority in residency, with junior residents having poorer outcomes. In addition, there was a statistically significant interaction effect on mental health outcomes, with moderate effect sizes between gender and working on the front line, as well as gender, working on the front line and seniority. Univariate analysis showed that the effect of gender on the depression score depended on working on the front line versus second line, and vice versa. Moreover, the effect of gender on all scores depended on working on the front line and seniority, with junior female front-line residents and male junior second-line residents exhibiting the poorest outcomes. Together, our findings present concerns about the psychological well-being of the junior residents during the COVID-19 pandemic.

In this study, a significant proportion of participants experienced symptoms of depression, anxiety and psychological symptoms. In a previous study during the acute SARS outbreak, 89% of healthcare workers who were in high-risk situations reported more negative psychological effects (using the Perceived Stress Scale) than the control group.^20^ During the COVID-19 pandemic in Wuhan, China, a survey among healthcare workers showed a high prevalence of depression (50.4%), anxiety (44.6%) and distress (71.5%).^6^ Similarly, 90% of the neurosurgery residents across different programmes believed that this pandemic had influenced their mental health and social life.^9^ In a national study among otolaryngology residents and attending physicians in the USA, the prevalence of depression, anxiety, distress and burnout among the medical residents were 10.6%, 47.9%, 60.2% and 21.8%, respectively. The authors also found that the residents had increased burnout relative to attending physicians.^12^ In terms of association with gender, previous studies have found a higher level of depression, anxiety, distress and burnout in women, which are probably explained by a combination of biological and social factors.^6,12^ In our study, female gender was not independently associated with any of the mental health outcomes. Nonetheless, our study demonstrated an interaction effect between gender and working on the front line, as well as an interaction between gender, working on the front line and seniority, which showed a moderate but significant effect on mental health outcomes. This probably means that female medical residents might be at a higher risk of detrimental psychological effects when they are junior residents and/or working on the front line, but are probably less affected as they gain experience (become senior residents) or as long as they are not deployed to COVID-19 sites. Possible explanations might include increased experiences of fear related to COVID-19 among women, but these fear experiences are probably attenuated by their medical experience.^21^

In contrast with previous findings, our study showed that residents did not report high levels of burnout, whereas the prevalence of burnout among the Romanian residents was as high as 76% during the COVID-19 pandemic.^13^ Internal medicine and psychiatry residents in Michigan, USA, have stated a lack of adequate personal protective equipment, physical and emotional exhaustion and overwhelming fear of catching the virus and infecting their loved ones, as potential grounds for burnout in front-line medical residents.^22^ They also suggested that the psychiatry residents, for example, who are not working on the front line may develop higher vicarious traumatisation, as suggested by a study in Wuhan illustrating that second-line nurses had significantly higher level of vicarious traumatisation than front-line nurses.^23^ Possible explanations for the lower propensity of developing burnout and vicarious traumatisation among the medical residents in Qatar are high levels of personal accomplishment and satisfaction with medicine, and a low level of depersonalisation, as illustrated in a cross-sectional study in 2017 among medical residents in the HMC.^24^ On the other hand, we do not have a direct comparison using scales used in this study. As part of ACGME-I accreditation, the well-being of the residents is monitored and HMC did not receive any citations regarding this in their 2018 site visit. Nonetheless, in general, medical residents experience exceptional stress during their training programmes because of long working hours, heavy workload, poor work environment, lack of social support, problems of relocation and difficult patients and families.^24^

Several studies have compared front-line and second-line healthcare workers, with the majority showing poorer mental health outcomes for those in high-risk environments.^25^ A recent study in Wuhan showed that working on the front line was an independent risk factor for worse mental health outcomes such as depression, anxiety, distress and insomnia.^6^ In our study, the male junior second-line residents and female junior front-line residents had the highest levels of psychological symptoms. A possible explanation could be that the junior residents have less clinical and infection control knowledge and experience, and consequently, are less prepared to face the current pandemic crisis.^25^ In Singapore, front-line medical residents had lower stress levels than second-line residents, because of the higher degree of psychological preparedness among front-line residents and anticipatory anxiety among second-line residents, despite the fact that second-line residents seem further away from the crisis.^11^ Furthermore, following the deployment of the residents to high-risk areas, second-line residents may have faced longer working hours, extra responsibilities and less supervision.

Our findings indicate worse mental health outcomes among junior residents. This is in accordance with the findings from a study in Wuhan, which indicated that healthcare workers with junior titles may be at higher risk of more severe mental health outcomes in terms of depression, anxiety, insomnia and distress.^6^ Nonetheless, a study in Singapore found no difference between the junior and senior residents in terms of psychological responses to the pandemic.^11^ On the other hand, the junior residents and second-line residents in Qatar were at increased risk for symptoms of depression, anxiety and stress. Consequently, their mental health may require special attention, bearing in mind that they are particularly vulnerable. In a recent paper, the leaders of the American Heart Association wrote that ‘residents and fellows — whether due to lack of experience, poor preparation or lack of training — are particularly vulnerable’. Therefore, they are the ones most in need of protection and mental health support during moments like this. They went on to state ‘Protect medical trainees on the COVID-19 front lines or do not send them in’.^26^ In France, a collaborative and helping crisis department has been set up for every medical resident in need. This department connects the residents in training with the psychiatry residents 24 hours a day, to address their mental health needs.^8^ It is also important for healthcare leaders to develop and implement support measures based on the needs and desires of the healthcare workers. For instance, in a survey among New York medical residents and other healthcare workers on potential wellness resources, self-guided counselling with access to a therapist, individual counselling and therapy, and an online clinician support group gathered the most interest.^16^

This study has several strengths, chief among them that the survey was sent to all medical residents in Qatar, and used validated, widely used questionnaires in the field. However, a few limitations need to be acknowledged. Indeed, we had a low response rate of 20% and the possibility of non-response bias cannot be ruled out. Indeed, it is possible that the nonrespondents were either too stressed to respond or were in good health and therefore not interested in this survey.^27^ Besides, our response rate is comparable with rates of 20% and 27.4% from national studies in USA and South Korea, respectively.^10,14^ This means that extrapolating our findings to the entire population of medical residents might be difficult. The observed power of 60.4% was also rather low, thus indicating the possibility of a type 2 error. Moreover, we did not include a control group and therefore are unable to definitely conclude that these symptoms in the medical residents differ from those of the general population. Last, but not least, we have not investigated secondary stressors, such as familial and social stressors, between the front-line and second-line residents, which may affect the residents’ longer-term mental health.^28^

In conclusion, our findings indicate that medical residents, and in particular the junior residents, display high levels of depressive, anxiety and stress symptoms during the COVID-19 pandemic. Contrary to preconceived ideas, these symptoms are not limited to frontline healthcare workers. Medical residents may require particular attention during a pandemic. This includes wellness resources such as individual counselling with a therapist, or a support group with the other residents. The reassuring professional quality-of-life scores in our study may indicate that the residents may benefit from engagement, opportunities for continuous education and other opportunities to grow in their position. Nonetheless, further studies are needed to capture the longitudinal picture during the ongoing COVID-19 pandemic, for it is important that institutions continue their efforts regarding the mental wellness of their medical residents.

## Data Availability

The data that support the findings of this study are available from the corresponding author, W.N.C.K., upon reasonable request and pending additional ethical approval.
